# Tumor cell-specific exon 3-deleted FOXP3 isoform promotes growth and metastasis via STAT3 in non-small cell lung cancer

**DOI:** 10.1093/lifemedi/lnag024

**Published:** 2026-07-07

**Authors:** Jia Peng, Yi Liu, Zhongqin Gong, Ming-Yue Li, Hao Jia, Xueqing Zhou, Calvin S H Ng, George G Chen, Shucai Yang

**Affiliations:** Department of Surgery, The Chinese University of Hong Kong, Prince of Wales Hospital, Hong Kong 00000, China; School of Ocean and Tropical Medicine, Guangdong Medical University, Zhanjiang 524023, China; Department of Surgery, The Chinese University of Hong Kong, Prince of Wales Hospital, Hong Kong 00000, China; GuangZhou Laboratory, Guangzhou International Bio Island, Guangzhou 510005, China; Department of Thyroid and Breast Surgery, Peking University Shenzhen Hospital, Shenzhen 518036, China; Department of Laboratory Medicine, Pingshan Hospital, Southern Medical University (People’s Hospital of Shenzhen Pingshan), Shenzhen 518118, China; Department of Surgery, The Chinese University of Hong Kong, Prince of Wales Hospital, Hong Kong 00000, China; Department of Surgery, The Chinese University of Hong Kong, Prince of Wales Hospital, Hong Kong 00000, China; Department of Otorhinolaryngology, Head and Neck Surgery, The Chinese University of Hong Kong, Prince of Wales Hospital, Hong Kong 00000, China; Department of Laboratory Medicine, Pingshan Hospital, Southern Medical University (People’s Hospital of Shenzhen Pingshan), Shenzhen 518118, China

**Keywords:** NSCLC, FOXP3 isoforms, oncogene, STAT3, chemoresistance

## Abstract

Forkhead box P3 (FOXP3), the master regulator of regulatory T cells (Tregs), is aberrantly expressed in tumor cells, including lung cancer. Alternative splicing generates multiple isoforms, among which the full-length FOXP3 (FOXP3FL) and exon 3-deleted variant (FOXP3Δ3) are predominant. We identified FOXP3Δ3 as the major isoform in non-small cell lung cancer (NSCLC) tumor cells. FOXP3Δ3 expression was elevated in tumor tissues and correlated with larger tumor size and advanced T stage. Functional assays showed that tumoral FOXP3Δ3 enhanced proliferation, invasion, migration, stemness, and apoptosis resistance in NSCLC cells, exerting stronger effects than FOXP3FL. Knockdown of FOXP3Δ3 in NSCLC cells suppressed tumor growth in nude mice, confirming its oncogenic activity *in vivo*. Mechanistically, FOXP3Δ3 upregulated phosphorylated STAT3 and mesenchymal markers (N-cadherin, Vimentin, Snail), while reducing epithelial marker E-cadherin compared with FOXP3FL. Coimmunoprecipitation indicated these differences may arise from distinct STAT3 binding affinities. Additionally, FOXP3Δ3 decreased cisplatin-induced apoptosis, suggesting a role in chemoresistance. In summary, FOXP3Δ3 is the predominant oncogenic FOXP3 isoform in NSCLC tumor cells, promoting tumor cell progression and therapy resistance through STAT3 signaling. These findings highlight FOXP3Δ3 as a potential therapeutic target.

## Introduction

Alternative splicing is a process by which different mRNA transcripts can be generated from a single gene. It allows the combination of different exons and introns during pre-mRNA processing, resulting in diverse protein isoforms with varied functions. Aberrant alternative splicing has been associated with various human diseases. As an example, Forkhead box P3 (FOXP3) exists in different isoforms that have distinct functions [1–4]. The major isoforms of FOXP3 generated by alternative splicing are FOXP3FL and FOXP3Δ3 in Tregs of *Homo sapiens* [5]. FOXP3Δ3 lacks exon 3, which is localized in the repressor domain, indicating it overlaps but also has a distinct function compared with the canonical full-length isoform. In Tregs, both FOXP3FL and FOXP3Δ3 are naturally expressed and exhibit a suppression ability in the tumor microenvironment (TME) [6, 7].

FOXP3 is not only the master regulator and the main marker of Tregs, but it is also expressed in tumoral cells in several types of cancer, including lung cancer [8]. In lung cancer, higher expression of FOXP3 has been associated with a poorer prognosis, indicating an oncogenic role [9–13]. However, these studies have examined the total length of FOXP3 rather than its specific isoforms. In other cancers, studies have also shown that FOXP3Δ3 is associated with a more aggressive phenotype and resistance to chemotherapy compared with FOXP3FL. For example, in thyroid cancer, the suppression of FOXP3Δ3 is associated with increasing cisplatin treatment effect [14]. In bladder cancer, abnormal expression of FOXP3Δ3 indicates poor overall survival and contributes to chemotherapy resistance. Interestingly, they also discovered that after exon sequencing, the expression level of FOXP3Δ3 is 6–9 times higher than FOXP3FL in two lung cancer cell lines [15]. This may suggest that FOXP3Δ3 may also be likely to play a crucial role in lung cancer, inspiring us to uncover the role of FOXP3Δ3 in lung cancer. To verify this hypothesis, we examined FOXP3Δ3 *in vivo* and *in vitro* to define its functional role and potential as a therapeutic target in lung cancer. In particular, we focus on the tumor cell-intrinsic functions of FOXP3Δ3 in non-small cell lung cancer (NSCLC), rather than its immunoregulatory roles in Tregs.

## Results

### FOXP3Δ3 is the major isoform of FOXP3 in NSCLC EGFR and KRAS mutations do not affect the status of FOXP3

**Figure 1.**
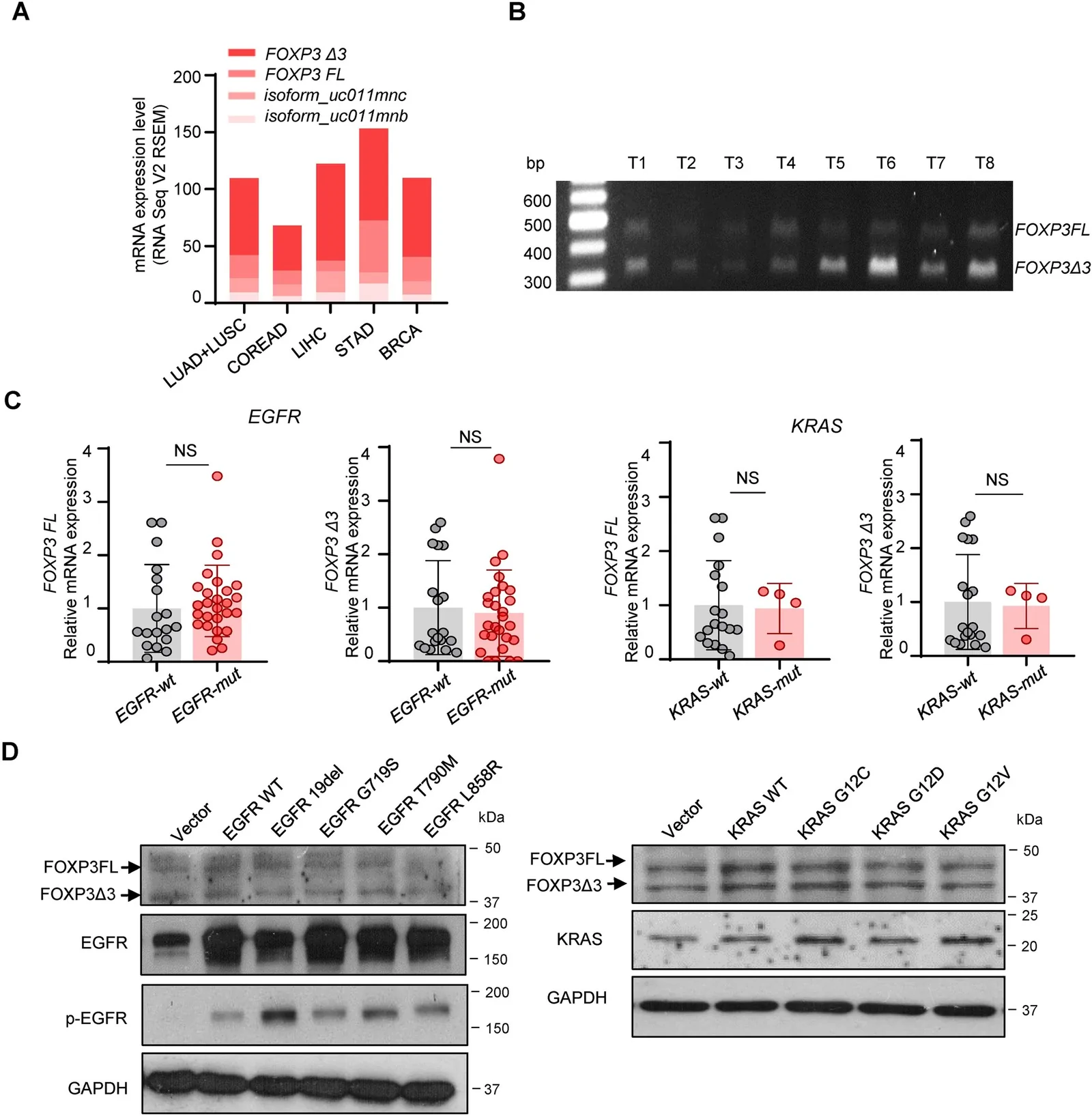
**FOXP3Δ3 is the major isoform of FOXP3, and EGFR and KRAS mutations do not affect the status of FOXP3 in NSCLC**. (A) FOXP3 isoforms mRNA expression level in Top5 most deadly cancers. (LUSC: lung squamous cell carcinoma, LUAD: lung adenocarcinoma, COREAD: colorectal adenocarcinoma, LIHC: liver hepatocellular carcinoma, STAD: stomach adenocarcinoma, BRCA: breast cancer.) Data were downloaded from TSVdb. (B) Gel electrophoretogram of the mRNA expression level of FOXP3FL and FOXP3Δ3 in NSCLC tumor samples. (C) The expression of FOXP3FL and FOXP3Δ3 in EGFR type (*n* = 36) and mutant (*n* = 33), KRAS type (*n* = 64) and mutant (*n* = 5) NSCLC patients’ clinical samples. (D) H1299 cells stably overexpressing different forms of EGFR and KRAS were harvested, and the expression of FOXP3FL and FOXP3Δ3 was determined by immunoblotting. Data are shown as mean ± SD. ns, not significant, by *t*-test.

To assess FOXP3 isoform usage in cancer, we analyzed four FOXP3 transcripts (*FOXP3FL*, *FOXP3Δ3*, *isoform_uc011mnb*, *isoform_uc011mnc*) across the five most lethal cancers in TCGA (TSVdb) [16] and found that FOXP3Δ3 is the predominant isoform in all five, accounting for more expression than the other variants combined (Fig. 1A). To further validate isoform distribution in lung adenocarcinoma (LUAD), we examined FOXP3 transcripts using GEPIA2 and UCSC Xena and confirmed that FOXP3-201 (FOXP3Δ3) and FOXP3-001 (FOXP3FL) are the two principal isoforms, whereas other variants show minimal or undetectable expression (Fig. S1A and S1B). Consistently, our in-house NSCLC cohort demonstrated that FOXP3Δ3 is also the dominant FOXP3 isoform in tumor tissues (Fig. 1B), indicating that FOXP3Δ3 is the major FOXP3 isoform in NSCLC.

Because EGFR and KRAS mutations are common and often biologically impactful in NSCLC [17], we examined whether they influence FOXP3 isoforms. Among 69 in-house NSCLC samples, EGFR mutations were detected in 47.8% of cases (L858R 20.3%, 19del 26.1%, plus five rare variants), and KRAS mutations in five cases (G12C, G12D, G12V, G12R). FOXP3Δ3 and FOXP3FL levels did not differ significantly between mutation-positive and wild-type tumors (Fig. 1C). In line with this, FOXP3 isoform expression was unchanged in H1299 cells engineered to express mutant or wild-type KRAS or EGFR (Fig. 1D), suggesting that FOXP3Δ3 and FOXP3FL status are largely independent of EGFR/KRAS mutation status.

### FOXP3Δ3 is overexpressed in NSCLC tissues compared with non-tumor tissues and correlates with poor clinical characteristics

**Figure 2.**
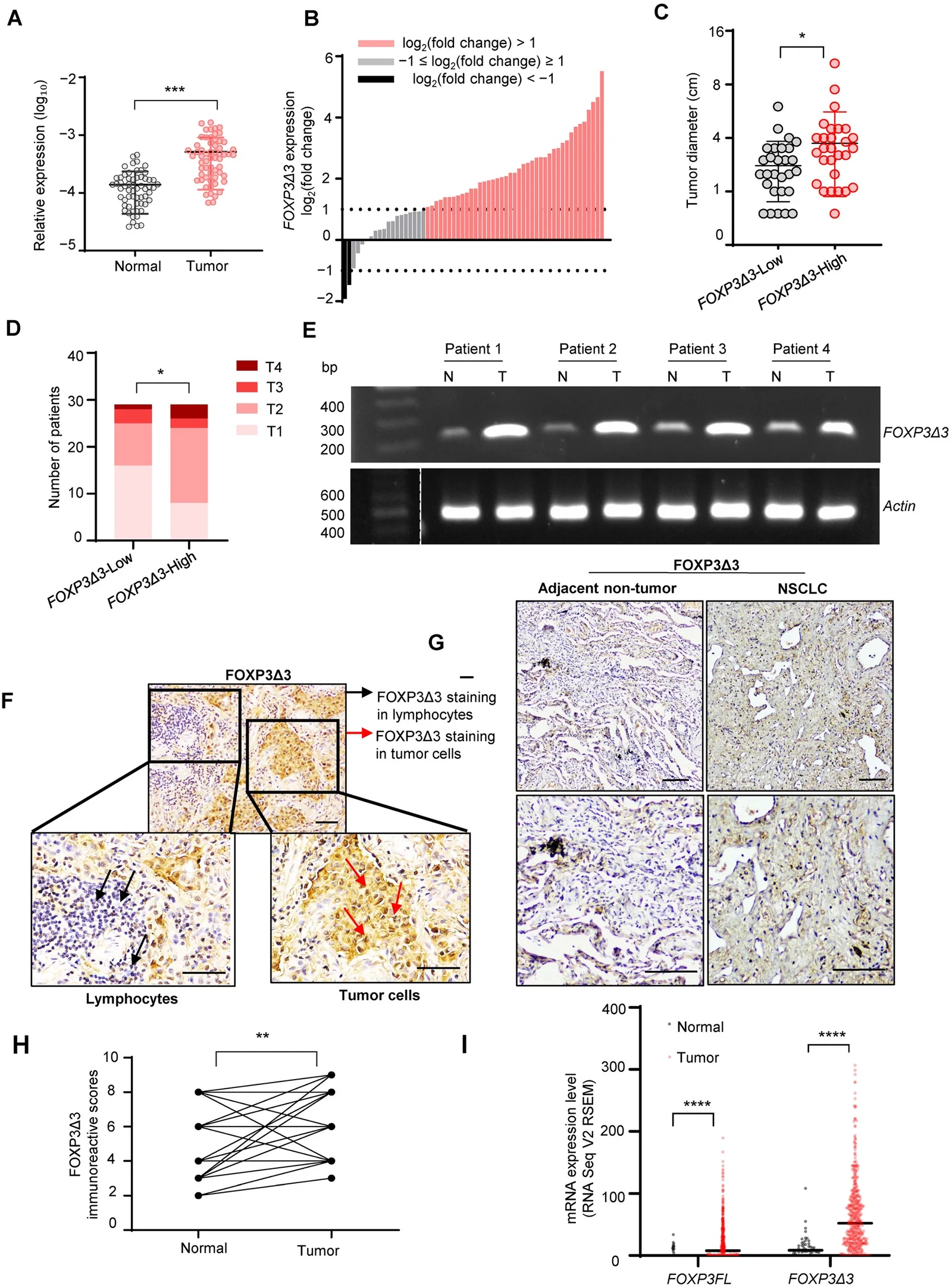
**FOXP3Δ3 is overexpressed in NSCLC tissues compared with non-tumor tissues and correlates with poor clinical characteristics**. (A) *FOXP3Δ3* mRNA expression level (by Q-PCR) in tumor tissues and paired adjacent non-tumor tissues in 60 pairs of in-house NSCLC patients’ samples. (B) log_2_(fold change) of *FOXP3Δ3* mRNA expression (by Q-PCR) in tumor tissues relative to paired adjacent non-tumor tissues from 60 in-house NSCLC patient samples. Red indicates ≥ 2-fold upregulation, black indicates ≥ 2-fold downregulation, and gray represents changes between −2- and +2-fold. The tumor size (C) and T-stage (D) in NSCLC patients express low and high levels of FOXP3Δ3. (E) Gel electrophoretogram showing amplified mRNA of FOXP3Δ3 in tumor tissue compared with adjacent non-tumor tissues in 4 pairs of NSCLC patients’ samples, β-Actin was used as an internal control. (F) Representative IHC images of FOXP3Δ3 staining in both lymphocytes and pneumonocytes in the tumor tissue of NSCLC patients. Black arrows point to lymphocytes, red arrows point to pneumonocytes. Scale bar, 100 μm. (G) Representative IHC images of FOXP3Δ3 staining in NSCLC tissues compared with adjacent non-tumor tissues (Sample No. 283). Scale bar 200 μm. (H) The IRS scores of FOXP3Δ3 levels in 47 pairs of NSCLC tissues and adjacent normal tissues were analyzed by paired *t*-test. (I) *FOXP3FL* and *FOXP3Δ3* mRNA expression levels in NSCLC tumor samples compared with the normal lung tissue samples in the TCGA database. Data were downloaded from TSVdb. Data are presented as mean ± SD. *P < 0.05, **P < 0.01, ***P < 0.001, by *t*-test.

Several studies have reported abnormal FOXP3 expression in lung cancer, but without distinguishing individual isoforms [3–7]. In our cohort, FOXP3Δ3 was identified as the major FOXP3 isoform in NSCLC. In 60 paired NSCLC samples, *FOXP3Δ3* mRNA was significantly higher in tumors than in matched non‑tumor tissues (*P *< 0.01; Fig. 2A and 2E), and 68.3% of cases showed more than a 2‑fold increase in FOXP3Δ3 in tumors versus adjacent non‑tumor tissues (Fig. 2B). These mRNA data were consistent with immunohistochemistry (IHC) from 100 NSCLC cases, which also demonstrated higher FOXP3Δ3 expression in the tumor compared with the adjacent lung (Fig. 2G and 2H). Although FOXP3Δ3 positivity was observed in both lymphocytes and pneumonocytes, these cell types are readily distinguished morphologically (small, round, high nuclear–cytoplasmic ratio versus larger, polygonal cells with lower nuclear–cytoplasmic ratio), allowing reliable assessment of tumor cell FOXP3Δ3 staining (Fig. 2F).

TCGA analysis further supported our findings, showing that the exon 3‑deleted FOXP3 isoform is the predominant isoform and is more highly expressed in tumors than in normal lung tissues (Fig. 2I). Together, these results indicate that FOXP3Δ3 is overexpressed in NSCLC at both mRNA and protein levels.

To assess clinical relevance, we examined the association between FOXP3Δ3 expression and clinicopathologic features. Patients were divided into high‑ and low‑expression groups according to the median FOXP3Δ3 level, and high FOXP3Δ3 expression correlated with larger tumor size (*t*-test, *P *= 0.023) and more advanced T stage (Mann–Whitney *U* test, *P *= 0.032; Fig. 2C and 2D; Table 1). In our in‑house NSCLC cohort, limited follow‑up precluded reliable isoform‑specific survival analysis. Therefore, we evaluated the FOXP3 exon‑skipping event ES89118 (corresponding to FOXP3Δ3) in UCSC Xena, which showed significant associations between FOXP3Δ3‑related splicing and survival outcomes in several other cancer types, including rectal adenocarcinoma (READ), sarcoma (SARC), and thyroid carcinoma (THCA) (Fig. S1C–E).

**Table 1.** Clinical characteristics of patients with NSCLC according to FOXP3Δ3 expression levels.

Characteristics		Total	High level	Low level	*P*-value
**Gender**	Male	33	16	17	0.795
	Female	27	14	13	
**Age (year)**		66.62 ± 7.05	66.40 ± 7.11	66.83 ± 7.23	0.816
**Smoking status**	NA	3	2	1	0.359
	Smoker	25	14	11	
	Non-smoker	32	14	18	
**Tumor diameter (cm)**		3.35 ± 1.67	6.13 ± 9.57	3.05 ± 1.42	0.023
**Stage**	NA	3	2	1	0.240
	I	30	12	18	
	II	11	7	4	
	III	15	9	6	
	IV	1	0	1	
**T status**	NA	2	1	1	0.032
	1	24	16	8	
	2	25	9	16	
	3	5	3	2	
	4	4	1	3	
**M status**	NA	2	1	1	1
	Positive	2	1	1	
	Negative	56	28	28	
**N status**	NA	2	1	1	1
	Positive	18	9	9	
	Negative	40	20	20	

A high level of FOXP3Δ3 was significantly associated with larger tumor size (*P *= 0.023) and advanced T status (*P *= 0.032).

### FOXP3Δ3 overexpression promotes proliferation, invasion, migration, and stemness in NSCLC cells

We have demonstrated that FOXP3Δ3 is the major isoform of FOXP3 and is overexpressed in NSCLC tumor tissues. But the functional role of FOXP3Δ3 in NSCLC remains unknown. To elucidate the biological function of FOXP3Δ3, stable FOXP3Δ3 overexpression and knockdown cell lines were established, respectively, according to the endogenous expression level of FOXP3.

**Figure 3.**
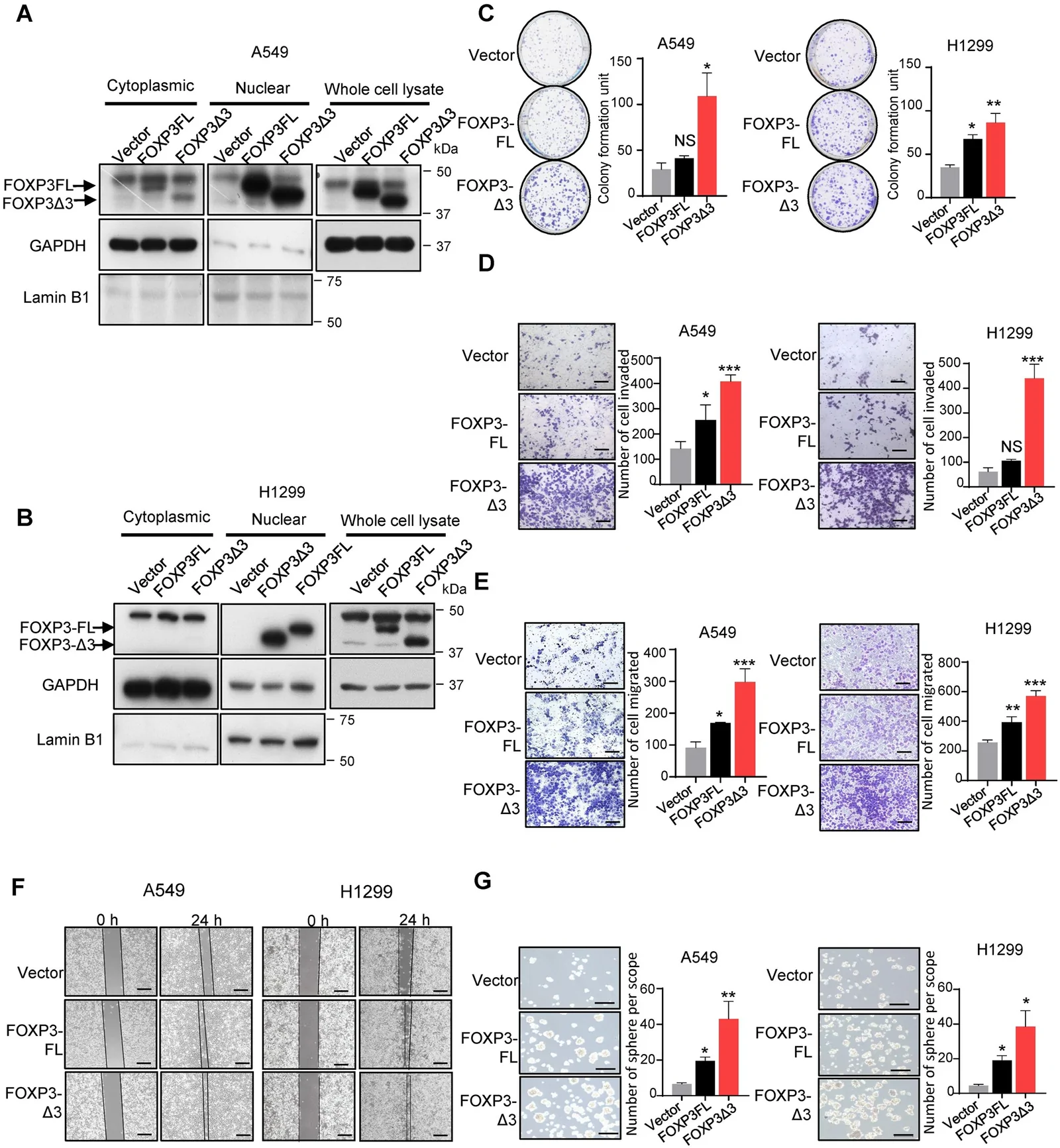
**FOXP3Δ3 overexpression promotes proliferation, invasion, migration, and stemness**. (A) A549 and (B) H1299 cells were stably overexpressed with FOXP3FL and FOXP3Δ3. After nuclear-cytoplasmic fractionation, the levels of FOXP3FL and FOXP3Δ3 were determined by immunoblot in the nucleus, cytoplasm, and whole cell lysate. Lamin B1 and GAPDH were used as nuclear and cytoplasmic internal controls, respectively. (C) In A549 and H1299 cells, the effect of FOXP3FL and FOXP3Δ3 on cell viability was evaluated by colony formation assay. Invasion (D) and migration (E) abilities were examined by the trans-well assay. Scale bar, 100 μm. (F) Migration ability was determined by a wound healing assay. Scale bar, 200 μm. (G) Stemness was determined by sphere formation assay. Scale bar, 200 μm. Data are presented as mean ± SD. *P < 0.05, **P < 0.01, ***P < 0.001 by one-way ANOVA. These experiments were repeated 3 times.

Stable FOXP3-overexpression NSCLC cell lines were established and transfection efficiency was determined by Western blot, as shown in Fig. 3A and 3B. FOXP3 is a transcription factor with a heterogeneous subcellular localization in both the nucleus and cytoplasm, but it can only exert its transcriptional function once translocated into the nucleus [8]. In this regard, the nuclear and the cytoplasmic fractions were isolated. It showed that FOXP3 was mainly in the nucleus (Fig. 3A and 3B).

After the establishment of stable FOXP3 overexpression cell lines, a colony formation assay was performed to investigate the cell proliferation ability, indicated by the counts of colonies formed. As plotted in Fig. 3C, FOXP3Δ3 could promote the proliferation of A549 (*P *< 0.05) and H1299 (*P *< 0.01). This promoting effect was modest in the FOXP3FL overexpression group in H1299 cells (*P *< 0.05) and was not detected in A549 cells.

In the next step, the trans-well assay and wound healing assay were employed for the purpose of detecting the invasion as well as the migration ability of FOXP3FL and FOXP3Δ3. Intriguingly, the forced expression of FOXP3Δ3 profoundly enhanced the invasion ability of A549 (*P *< 0.001) and H1299 (*P *< 0.01) according to the number of cells invaded into the low chamber of the inserts in the trans-well assay (Fig. 3D). While in the FL group, the effect was negligible in H1299 cells. Cell migration ability was examined by trans-well assay (Fig. 3E) and wound healing assay (Fig. 3F), the width of wounds and cells invaded into the low chamber suggested a stronger migration ability in the FOXP3Δ3 overexpression group compared with the canonical isoform group.

Besides, in the sphere formation assay, the increasing number of spheres formed by FOXP3Δ3 indicated that FOXP3Δ3 could facilitate the stemness of NSCLC A549 and H1299 cells (*P *< 0.05) compared with cells with FOXP3FL overexpression (Fig. 3G).

### Downregulation of tumoral FOXP3Δ3 inhibits proliferation, invasion, migration, and tumor growth

**Figure 4.**
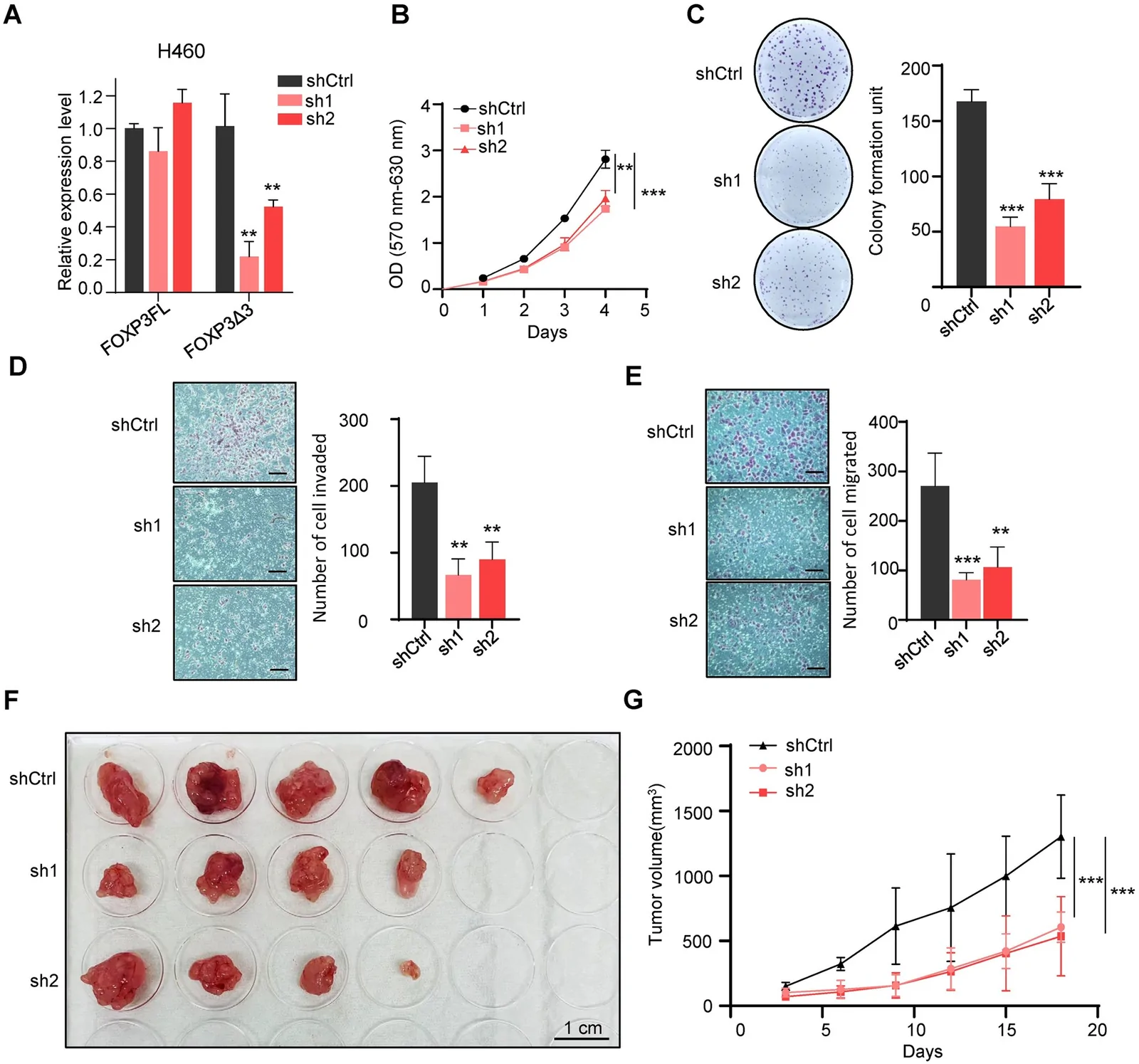
**Downregulation of FOXP3Δ3 inhibits proliferation, invasion, migration, and tumor growth in the subcutaneous nude mouse model**. (A) *FOXP3Δ3* and *FOXP3FL* mRNA expression levels in FOXP3Δ3-knockdown H460 cells were validated by qPCR. In H460 cells, the effect of FOXP3Δ3 knockdown on cell viability was evaluated by MTT assay (B) and colony formation assay (C). Invasion (D) and migration (E) abilities were examined by trans-well assay. Scale bar, 100 μm. (F) Representative image of tumors, scale bar, 1 cm. (G) Tumor growth curves of xenograft tumors derived from subcutaneous injection of H460-shCtrl (*n* = 5), H460-FOXP3Δ3sh1 (*n* = 4), and H460-FOXP3Δ3sh2 (*n* = 4) cells. Data are presented as mean ± SD. **P < 0.01, ***P < 0.001 by two-way ANOVA.

Given the potent effects of FOXP3Δ3 overexpression on proliferation, invasion, migration, and stemness, we next assessed the impact of FOXP3Δ3 knockdown. FOXP3Δ3 was silenced in H460 cells using two lentiviral shRNAs (FOXP3Δ3‑sh1, FOXP3Δ3‑sh2), with pLKO.1 as control (shCtrl), and efficient isoform‑specific knockdown was confirmed by qPCR (Fig. 4A). FOXP3Δ3 depletion significantly inhibited cell proliferation in MTT assays (Fig. 4B) and reduced both the number and size of colonies in colony formation assays (Fig. 4C). In addition, FOXP3Δ3 knockdown markedly suppressed invasion and migration in trans‑well assays (Fig. 4D and 4E).

Based on the results of *in vitro* studies that FOXP3Δ3 depletion could inhibit cell growth, we examined whether the knockdown of FOXP3Δ3 could inhibit tumorigenicity *in vivo* in a nude mouse subcutaneous tumor model. Three types of stable FOXP3Δ3-knockdown H460 cells (5 × 10^6^): FOXP3Δ3-sh1 (sh1), FOXP3Δ3-sh2 (sh2), and shCtrl were inoculated into the right flank of 4-week-old female BALB/c nude mice (*n *= 5/group). The length and width of the tumor were measured every 3 days, and mice were sacrificed on the 18th day after injection. The volume of the tumors was calculated by 1/2 (length × width × width) (Fig. 4F). As plotted in the tumor growth curve, the FOXP3Δ3 knockdown groups, sh1 (*P *< 0.001) and sh2 (*P *< 0.001) (Fig. 4G), exhibited a large reduction in the growth rate compared with the shCtrl group. These results suggested that FOXP3Δ3 knockdown could suppress tumor growth *in vivo*.

### STAT3 is a downstream target of FOXP3Δ3

**Figure 5.**
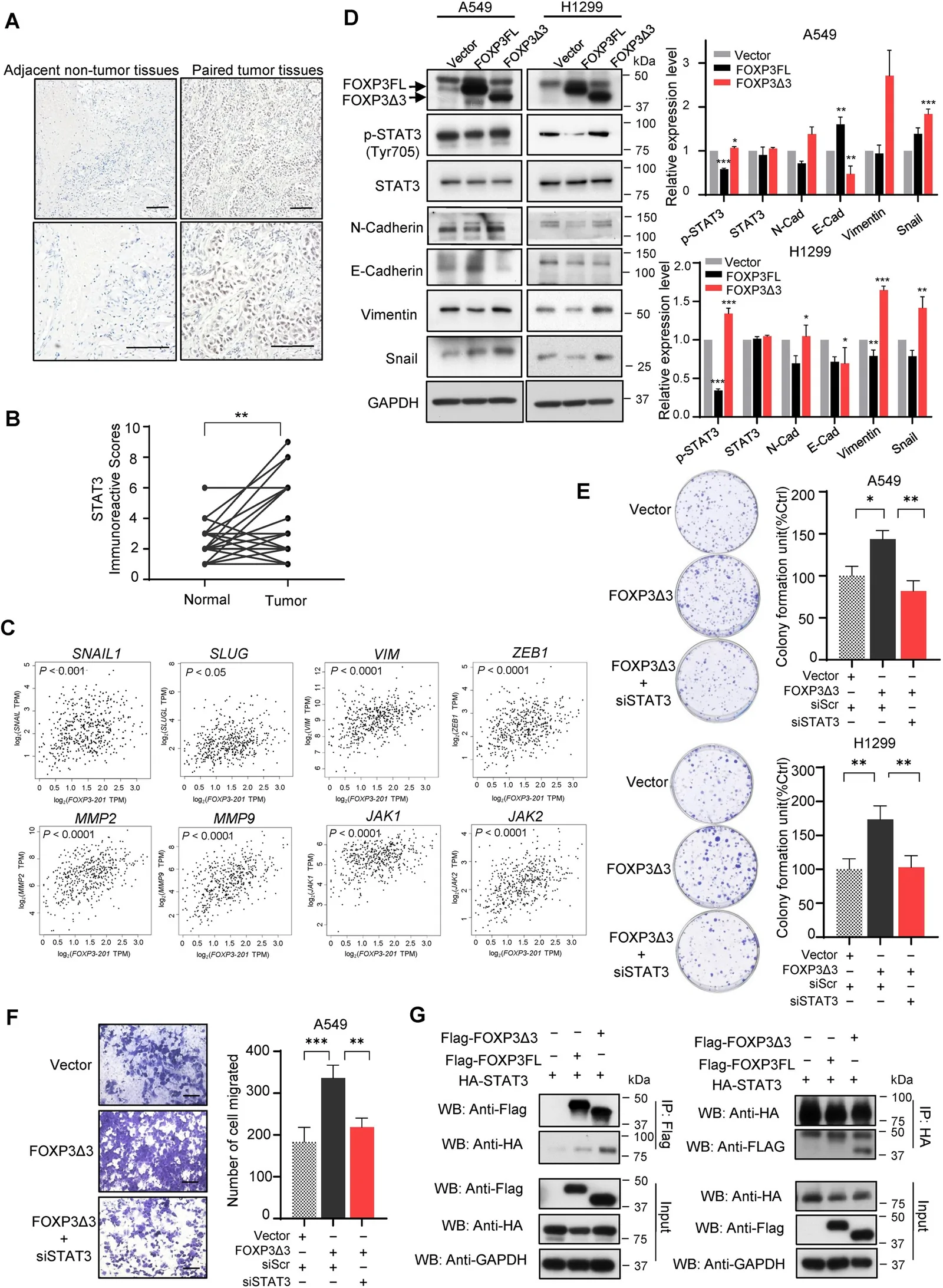
**STAT3 is a downstream target of FOXP3Δ3**. (A) Representative IHC images of p-STAT3 (Tyr705) staining in NSCLC tissues compared with adjacent non-tumor tissues. Scale bar, 200 μm. (B) The IRS scores of p-STAT3 levels in 46 pairs of NSCLC tissues and adjacent normal tissues were analyzed by paired *t*-test. (C) The correlation of FOXP3Δ3 and EMT-related STAT3 downstream targets: Vimentin (*P* < 0.0001), Snail (*P* < 0.001), Slug (*P* < 0.05), ZEB1 (*P* < 0.0001), MMP2 (*P* < 0.0001), MMP9 (*P* < 0.0001), JAK1 (*P* < 0.0001), and JAK2 (*P* < 0.0001) based on TCGA database (by GEPIA2: gepia.cancer-pku.cn). (D) The protein expression levels of p-STAT3, Vimentin, and Snail were determined by immunoblot, images were quantified by ImageJ, and grayscale values were plotted in a bar graph. *n* = 3. (E) Cell viability of A549 and H1299 cells transfected with FOXP3Δ3 overexpression plasmid or FOXP3Δ3 overexpression plasmid plus STAT3 siRNA was assessed by colony formation assay. *n* = 3. (F) Migration ability was evaluated by trans-well assay in A549 cells. *n* = 3, scale bar, 100 μm. (G) Interaction between FOXP3FL/Δ3 and STAT3 was examined by coimmunoprecipitation assay in HEK-293T cells. Data are presented as mean ± SD. *P < 0.05, **P < 0.01, ***P < 0.001 by one-way ANOVA. These experiments were repeated 3 times.

STAT3, a transcription factor, acts as an oncogene in various cancers [18, 19]. In lung cancer, p-STAT3 was reported to be an oncogene and activated in most NSCLC patients and cell lines [20–24]. In this study, we tested the expression of p-STAT3 protein in 46 pairs of NSCLC patient specimens using the IHC method. We found that the expression of p-STAT3 was upregulated in lung tumor tissues compared with adjacent normal lung tissues, indicated by the deep staining of the nuclear p-STAT3 (Fig. 5A and 5B).

p-STAT3 was reported to promote metastasis in lung cancer lines [20–24]. We analyzed the correlation of FOXP3Δ3 and STAT3 downstream targets based on the TCGA database and found that FOXP3Δ3 was positively correlated with some EMT-related STAT3 downstream targets: Vimentin, Snail, Slug, ZEB1, MMP2, and MMP9 (Fig. 5C). We further analyzed key upstream components of the JAK–STAT pathway, including JAK1 and JAK2. Both JAK1 and JAK2 showed significant positive correlations with FOXP3 expression (Fig. 5C). These results support potential crosstalk between FOXP3Δ3 and the JAK–STAT signaling axis. To consolidate these findings, a Western-blot assay was conducted to detect the expression of relevant proteins. We found that the overexpression of FOXP3FL modestly suppressed STAT3 phosphorylation and the expression of downstream mesenchymal markers, whereas FOXP3Δ3 did not exhibit a comparable inhibitory effect. In A549 cells, FOXP3FL did not appreciably reduce Snail, while clearer inhibitory effects were observed for N-cadherin and Vimentin, suggesting cell-line-dependent differences in EMT signaling regulation. As a result, STAT3 signaling appeared relatively enhanced in FOXP3Δ3-expressing cells when directly compared with FOXP3FL, rather than showing a strong absolute increase over control (Fig. 5D). This finding could at least account for the functional differences between the two isoforms. To verify this finding, colony formation and trans-well assays were performed to detect the impact of STAT3 on proliferation and migration abilities. We found that the downregulation of STAT3 by siRNA could largely reduce the proliferation and migration of NSCLC cells caused by FOXP3Δ3 (Fig. 5E and 5F).

To gain insights into the mechanism of how STAT3 and FOXP3 interact, a coimmunoprecipitation assay was performed to detect the protein–protein interaction between STAT3 and FOXP3. HEK293T cells were transfected with different forms of FOXP3 plasmids (Vector, Flag-FOXP3FL, Flag-FOXP3Δ3) and HA-STAT3 plasmids. As shown in Fig. 5G, less HA-STAT3 was detected to be pulled down by Flag beads in the FOXP3FL group compared with the FOXP3Δ3 group (left). Consistently, less FOXP3FL protein was pulled down by HA beads (right). These results indicate that exon 3 may have a negative impact on the binding between FOXP3 and STAT3, and that the different binding ability may at least partly explain the different phosphorylation levels of STAT3.

### FOXP3Δ3 and chemoresistance in NSCLC cells

**Figure 6.**
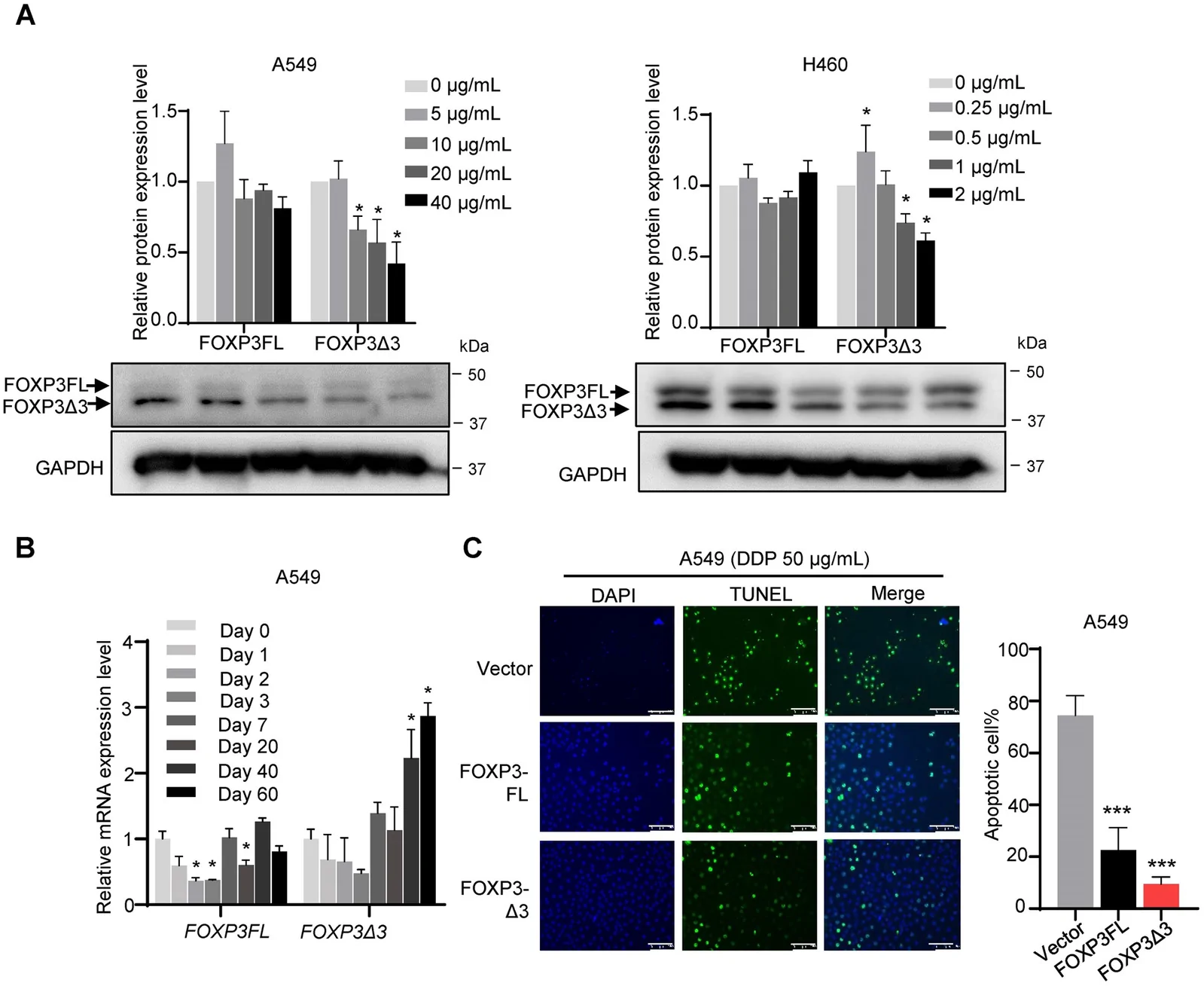
**FOXP3Δ3 and cisplatin (DDP) treatment**. (A) A549 and H460 cells were treated with cisplatin at the indicated dosage for 48 h, FOXP3Δ3 and FOXP3FL protein expression levels were determined by immunoblot. (B) A549 cells were treated with cisplatin (starting from 1 μg/mL, slowly increasing the cisplatin concentration by 1–2 µM/week) for 60 days to induce cisplatin resistance. *FOXP3Δ3* mRNA expression level evaluated by qPCR, *n* = 3. (C) A549 cells expressing different forms of FOXP3 were treated with 50 μg/mL cisplatin for 24 h, apoptotic rate was examined by TUNEL assay. Scale bars, 50 µm. Data are presented as mean ± SD. *P < 0.05, ***P < 0.001 by one-way ANOVA.

After being treated with increasing doses of cisplatin, a gradual decrease of FOXP3Δ3 was detected in both A549 and H460 cell lines (Fig. 6A). However, the expression of FOXP3FL was not affected. Moreover, an obvious increase of FOXP3Δ3 was detected in a prolonged period of cisplatin treatment in A549 cells (Fig. 6B). Furthermore, the TUNEL assay showed that FOXP3Δ3 could reduce apoptosis induced by cisplatin (Fig. 6C), whereas the anti-apoptosis ability of FOXP3FL was much weaker. These results suggest that FOXP3Δ3, rather than the full-length isoform, may play a role in cisplatin treatment. The prolonged period of cisplatin treatment may cause chemoresistance by inducing FOXP3Δ3.

**Figure 7.**
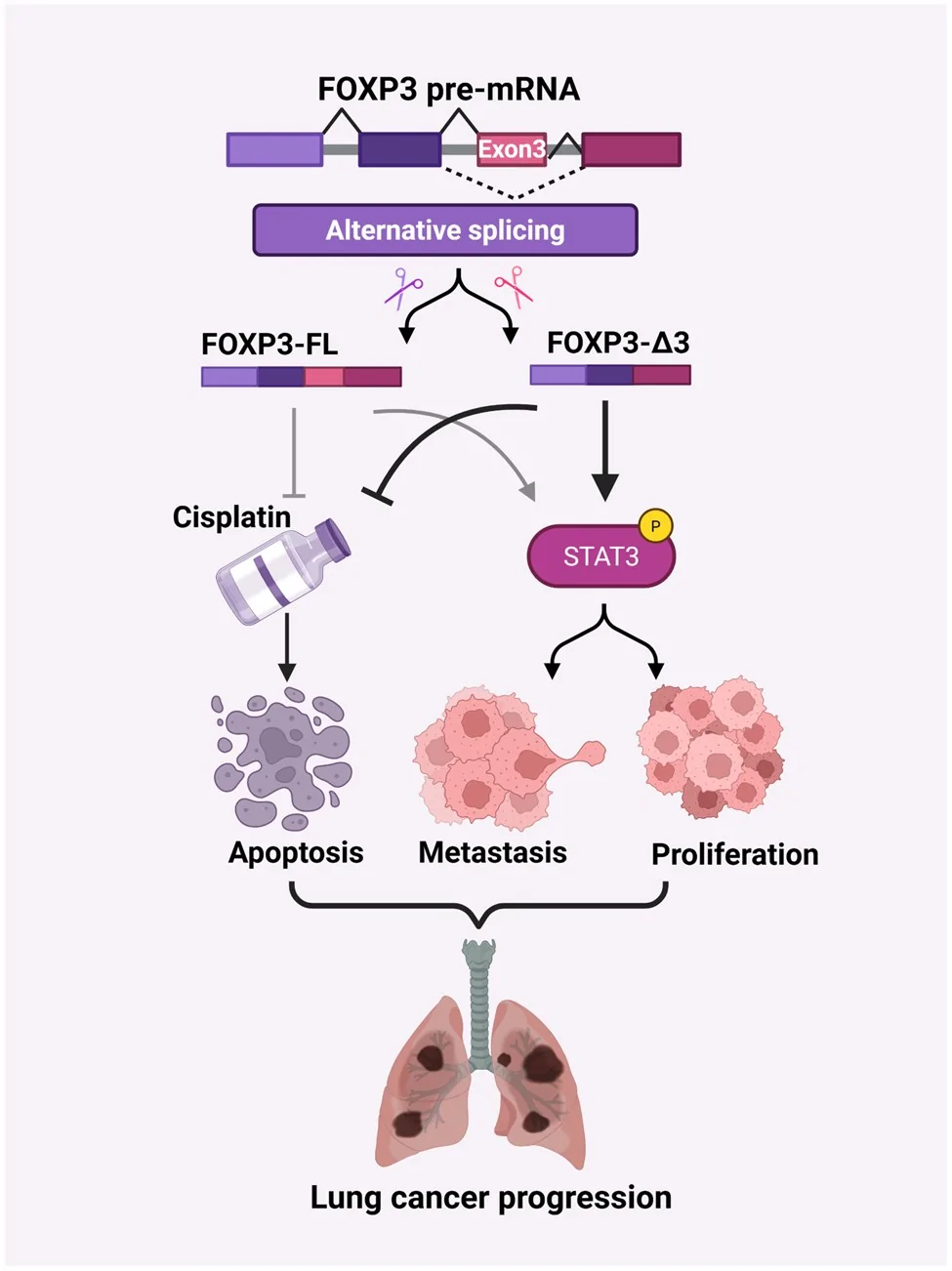
**Graphical abstract**. FOXP3Δ3, an isoform of FOXP3, is upregulated in NSCLC tissues, enhancing tumor growth, invasion, migration, and chemoresistance. It modulates p-STAT3 levels and epithelial–mesenchymal transition markers, with stronger effects compared to FOXP3FL. Knockdown studies confirm FOXP3Δ3’s role in tumor progression, suggesting its potential as a therapeutic target in NSCLC. Created in BioRender. Liu, X. (2026) BioRender. com/2fboz4z.

Collectively, our study reveals that FOXP3Δ3, a major isoform of FOXP3, is markedly upregulated in NSCLC tissues, where it plays a pivotal role in promoting tumor growth, invasion, and migration, while simultaneously reducing apoptosis and enhancing chemoresistance. This isoform significantly boosts the oncogenic potential of NSCLC by upregulating phosphorylated STAT3 and mesenchymal markers, such as N-cadherin, Vimentin, and Snail, and by downregulating the epithelial marker E-cadherin. This effect is attributed to differential binding abilities between STAT3 and the various FOXP3 isoforms, a relationship further elucidated through coimmunoprecipitation assays. Importantly, knockdown of FOXP3Δ3 *in vivo* suppresses tumor development, underscoring its potential as a therapeutic target to combat NSCLC and overcome associated chemoresistance (Fig. 7).

## Discussion

Unlike murine in which FOXP3 only exists in one form, FOXP3 can generate at least 5 variants through alternative splicing in humans: FOXP3FL, FOXP3Δ3, FOXP3Δ8, FOXP3Δ3,4, and FOXP3Δ3,8 [1–3, 25]. Among these isoforms, FOXP3FL and FOXP3Δ3 make up approximately 95% of FOXP3 expression in Tregs [5]. Jia et al. [2] have demonstrated that FOXP3Δ3 is the dominant isoform expressed in 31 types of cancers in the TCGA database. This exon 3 deleted isoform is also correlated with poor prognosis and is the major isoform contributing to the chemoresistance in bladder cancer [15]. Here, we demonstrated that FOXP3Δ3 was the major isoform in our in-house NSCLC patients’ tumor samples as well as NSCLC cell lines. This finding was further confirmed by analyzing the TCGA database.

It is well known that driver mutations commonly occur in NSCLC, and EGFR and KRAS mutations are the most notorious [26]. Therefore, we examined whether they influence FOXP3 isoforms. FOXP3Δ3 and FOXP3FL levels did not differ between EGFR/KRAS mutant and wild‑type tumors, and isoform expression was unchanged in EGFR‑ or KRAS‑engineered H1299 cells, indicating that FOXP3FL/FOXP3Δ3 status is largely independent of EGFR/KRAS mutation status. This supports the use of A549, H1299, and H460 cells for FOXP3 gain‑ and loss‑of‑function studies regardless of their driver mutation status.

Although FOXP3 has been shown to be aberrantly expressed in lung cancer, previous studies have failed to examine the specific isoforms [11, 13, 27, 28]. Here, we demonstrated that the expression of FOXP3Δ3 was upregulated in NSCLC tissues compared with adjacent non-tumor tissues with the aid of IHC and qPCR methods. Since FOXP3 is not only an oncogene in NSCLC cells but also a master regulator in Tregs, its expression is not restricted to tumoral cells. Thus, it is important to distinguish the cell types in which FOXP3 expresses is important when studying its expression in cancer tissues. In our IHC study of NSCLC tissues, we noted that FOXP3Δ3 was positive in both lymphocytes and pneumonocytes. Fortunately, these two types of cells can be easily distinguished by cell morphology. Therefore, IHC can still be a reliable approach to examining the expression of FOXP3Δ3 in NSCLC tissues.

In NSCLC, accumulating evidence indicates that FOXP3 exerts tumor-promoting effects in both compartments: in tumor cells, FOXP3 enhances proliferation, metastasis, cancer stemness, and drug resistance, while in the immune microenvironment, FOXP3-expressing Tregs mediate immunosuppression and facilitate immune escape [29]. Therefore, unlike some context-dependent regulators that exert opposing roles in tumor versus immune cells, FOXP3 appears to function in a convergent, tumor-promoting manner in lung cancer. Although the present study focuses on the tumor cell-intrinsic role of FOXP3Δ3, this is indeed a limitation of our study. Incorporating direct analyses of FOXP3 wild‑type and splice variants in Tregs, as well as tumor-immune crosstalk models, will be an important direction for future work to complement the tumor cell-centered findings reported here. However, incorporation of the immune context would be expected to further strengthen, rather than weaken, our conclusions. From a therapeutic perspective, targeting FOXP3 may thus provide a dual benefit by simultaneously suppressing tumor cell aggressiveness and alleviating Treg-mediated immunosuppression, offering a strategy to combat NSCLC by killing two birds with one stone.

Clinically, high FOXP3Δ3 expression in our cohort was associated with larger tumor size and advanced T stage, but not with distant and lymph node metastasis, which is inconsistent with the result reported by Li et al. [30]. This inconsistency might be due to the bias of the cohort. For example, patients with distant or lymph node metastasis were not eligible for surgery in our clinics, so we were unable to have these samples in our study. The limited number of samples may also be a factor. The high level of FOXP3 was demonstrated to predict poor overall survival and recurrence-free survival in NSCLC [28]. However, FOXP3Δ3 failed to have a significant impact on the overall survival in our study (data not shown). Although a definitive association between FOXP3Δ3 expression and survival could not be established in our NSCLC cohort, exploratory analysis using UCSC Xena revealed that FOXP3Δ3-associated exon skipping was significantly correlated with survival outcomes in multiple other cancer types. These findings support the broader biological and potential clinical relevance of FOXP3Δ3, while highlighting the need for larger NSCLC cohorts with complete survival data to validate its prognostic value in lung cancer.

Several limitations related to cohort size should be noted. The modest number of cases used for certain analyses may limit statistical power. Owing to the retrospective nature of our in-house cohort and current limitations in sample availability, further expansion of these cohorts was not feasible in the present study. In addition, limited follow-up information restricted definitive survival analysis in NSCLC. These constraints collectively highlight the need for larger, independent, and prospectively collected cohorts to further validate the clinical and molecular significance of FOXP3Δ3 in future studies.

In recent years, increasing evidence has shed light on the role of FOXP3 in lung cancer. FOXP3 was identified to drive proliferation of NSCLC by regulating the transcription activity of GINS1 [12]. The expression of FOXP3 was positively correlated with proliferation marker Ki-67, and the downregulation of FOXP3 showed a suppressive effect on the proliferation of LUAD [30]. FOXP3 was reported to enhance NSCLC cell proliferation via upregulating the level of CCND1 [27]. Another research also showed that FOXP3 could promote invasion and migration of NSCLC cells via activating Notch1-Hes1 pathway and regulating EMT [13]. Moreover, FOXP3 could promote cell proliferation, invasion, and EMT by Wnt signaling pathway [28]. FOXP3 could also stimulate CSC via activating the Notch signaling pathway [11, 26]. All these studies have suggested the positive role of FOXP3 in enhancing proliferation, promoting metastasis, and stimulating CSC. In our functional studies, FOXP3Δ3 was demonstrated to promote proliferation, invasion, migration, induce CSC, and reduce apoptosis of NSCLC cells, and it showed a stronger effect compared with the canonical isoform FOXP3FL. In addition, FOXP3Δ3 knockdown suppressed tumor growth in a subcutaneous nude mouse model, which further consolidates it as an oncogenic molecule in NSCLC. To the best of our knowledge, this is the first report to study FOXP3Δ3 details in NSCLC.

Emerging evidence indicates that the biological functions of FOXP3Δ3 exhibit strong isoform and tissue-dependent characteristics. In hepatocellular carcinoma, full-length FOXP3 and FOXP3Δ3 are the dominant isoforms. However, tumor suppressive activities, including growth inhibition, reduced migration, and induction of apoptosis via c-Myc repression, are largely attributed to full-length FOXP3, whereas FOXP3Δ3 displays attenuated or neutral effects. Thus, exon 3 loss in HCC appears to weaken tumor suppressive transcriptional programs [31]. In contrast, in bladder cancer, tumor cell FOXP3, particularly FOXP3Δ3, enhances HIF-1α protein stability, upregulates VEGF and glucose transporter genes, correlates with advanced tumor stage, chemotherapy resistance, and reduced intratumoral CD8^+^ T-cell infiltration [15, 32]. Thyroid cancer studies likewise suggest that FOXP3 splice variants participate in a complex regulatory network with context-dependent oncogenic effects [33].

Across cancer types, the balance between full‑length FOXP3 and FOXP3Δ3 appears to be highly context‑dependent: in hepatocellular carcinoma, tumor‑suppressive functions are largely attributed to FOXP3FL, whereas FOXP3Δ3 shows attenuated or neutral effects, while in bladder, thyroid cancer, and NSCLC, FOXP3Δ3 is more consistently linked with aggressive behavior, EMT, and therapy resistance.

Mechanistically, exon 3 encodes part of the N-terminal regulatory domain of FOXP3, the repressor domain, that governs protein–protein interactions and transcriptional regulation. Its deletion may reshape cofactor recruitment and partially release intramolecular repression while preserving DNA binding, thereby shifting FOXP3 toward cooperation with oncogenic pathways such as STAT3, HIF-1α, and EMT regulators [34]. This model provides a plausible explanation for why exon 3 deletion can confer distinct, tissue-specific functional properties on FOXP3Δ3, although detailed structure-function and interactome mapping will be needed in future studies to validate these hypotheses. In NSCLC, where hypoxia adaptation, EMT, and metabolic reprogramming are dominant selective pressures, this structural reprogramming likely favors the retention of FOXP3Δ3 as a tumor-promoting isoform. Together, these observations support a model in which exon 3 deletion converts FOXP3 into a context-dependent oncogenic coregulator, explaining the distinct functional properties of FOXP3Δ3 across tumor types.

STAT3 is originally identified as a key mediator of the IL-6 signaling pathway and a core component of the JAK–STAT pathway. It acts as a transcription factor in the downstream of various cytokines, growth factors, and interferons. A number of studies have demonstrated that STAT3 is an oncogene [18, 19]. The majority of studies have shown that STAT3 is activated in most NSCLC patients and cell lines, that the level of activated STAT3 (p-STAT3) is correlated with advanced stage [19], and that it promotes metastasis [20–24]. In this study, we found that the expression of p-STAT3 protein was upregulated in lung tumor tissues compared with adjacent normal lung tissues, which is consistent with previous studies [18, 19]. Furthermore, we found that FOXP3Δ3 was positively correlated with some EMT-related STAT3 downstream targets: Vimentin, Snail, Slug, ZEB1, MMP2, and MMP9. Western-blot assay also revealed that FOXP3Δ3 upregulated the expression of p-STAT3 and downstream mesenchymal markers (N-cadherin, Vimentin, snail), while downregulated epithelial marker (E-cadherin). However, FOXP3FL presented modest or even opposite effects. These findings provide a mechanistic basis for the functional divergence between FOXP3Δ3 and FOXP3FL.

Mailer et al. [35] indicated that p-STAT3 acted as a cofactor of FOXP3 through binding to its 71–105 amino acid site, which is precisely where the repressor domain is located. Interestingly, our coimmunoprecipitation assays revealed that FOXP3Δ3 exhibits stronger binding to STAT3 than FOXP3FL, indicating a distinct regulatory relationship. The absence of exon 3 may alter FOXP3 protein conformation or the accessibility of STAT3-binding surfaces, thereby facilitating enhanced STAT3 association and activation. Nevertheless, structure–function validation using STAT3 mutants or domain-mapping approaches will be required to define the precise molecular basis of this interaction. Due to current funding and resource limitations, these experiments could not be performed in the present study and therefore represent a limitation.

Importantly, our expanded TCGA-based analyses further indicate that FOXP3Δ3 participates in broader signaling network crosstalk, including EMT regulation and upstream JAK–STAT signaling, as supported by significant correlations with EMT regulators as well as JAK1 and JAK2. Collectively, these findings indicate that STAT3 represents one important, but not exclusive, downstream mediator of FOXP3Δ3, and that additional signaling pathways likely cooperate to drive its oncogenic functions.

Together with our EMT‑marker profiling, TCGA‑based correlations with JAK1/JAK2 and multiple STAT3 targets, and STAT3 knockdown rescue experiments, these data support STAT3 as an important mediator of FOXP3Δ3‑driven malignant phenotypes in NSCLC, but not as its sole effector pathway. We acknowledge that the broader signaling network downstream of FOXP3Δ3, including additional EMT and survival pathways, was not systematically mapped in this study, and we now explicitly highlight comprehensive pathway‑level analyses as an important direction for future work when additional resources are available.

Cytotoxic chemotherapy remains an important component of systemic therapy for most patients [36]. Previous studies have shown that FOXP3 is an oncogene in NSCLC [10–13], and our results further implicate FOXP3Δ3 as the isoform most closely linked to these malignant traits. Our cisplatin experiments showed that FOXP3Δ3, but not FOXP3FL, is dynamically modulated by cisplatin exposure and that FOXP3Δ3 overexpression reduces cisplatin‑induced apoptosis, suggesting that FOXP3Δ3 may contribute to acquired chemoresistance. This finding is supported by a study in bladder cancer, in which the overexpression of FOXP3Δ3 results in acquired resistance to cisplatin and gemcitabine treatment [15]. While our *in vitro* cisplatin-induced apoptosis assays support a role for FOXP3Δ3 in chemoresistance, the lack of *in vivo* chemoresistance models and patient-derived xenograft validation represents an important limitation. Therefore, the therapeutic relevance of FOXP3Δ3 in chemotherapy response should be interpreted with caution and requires further validation in future *in vivo* studies.

In conclusion, our study has demonstrated that FOXP3Δ3 is the major isoform of FOXP3 and is overexpressed in NSCLC. FOXP3Δ3 can not only promote tumor growth and metastasis but also inhibit the apoptosis induced by cisplatin. These results suggest that as an oncogene, FOXP3Δ3 may be a potential therapeutic target to overcome chemoresistance in NSCLC.

### Research limitations

This study has several limitations. First, the sample size and clinical cohort were relatively modest, which may limit the generalizability of our findings, particularly regarding IHC staining. Second, although FOXP3Δ3 expression was validated in patient tissues and functional assays, we did not perform lineage-tracing experiments to definitively distinguish tumor-derived FOXP3Δ3 from immune cell expression. Third, while STAT3 was identified as a downstream target, the broader signaling network and possible crosstalk with other oncogenic pathways were not fully explored. Finally, our cisplatin-based experiments suggest a role in chemoresistance, but validation with additional agents like cisplatin‑treated xenografts, patient‑derived xenografts, and in clinical settings is warranted.

## Methods

### Research ethics

Human studies were approved by the Joint Chinese University of Hong Kong–New Territories East Cluster Clinical Research Ethics Committee (Joint CUHK-NTEC CREC) in advance, CREC Ref No. 2021.011. All the patients had signed an informed consent form. Animal experiments were approved by the Animal Ethics Committee of the Chinese University of Hong Kong, AEC Ref No. 21-037-GRF.

### Clinical samples

A total of 108 patients diagnosed with NSCLC and underwent surgical operation in the Department of Surgery in Prince of Wales Hospital between 2004 and 2018 were included in this study. All the patients had signed an informed consent form and ethics approval was also obtained from the joint Chinese University of Hong Kong–New Territories East Cluster Clinical Research Ethics Committee in advance.

### Immunohistochemistry analysis

Paraffin blocks were cut into a thickness of 5 μm and attached to an adhesive slide, followed by deparaffinization in xylene and rehydration with ethanol. Antigen retrieval was done using sodium citrate buffer (pH 6.0) and blocked with 2.5% horse serum for 30 min. Primary antibodies were incubated overnight at 4°C, followed by peroxidase inactivation using 3% H_2_O_2_. Secondary antibodies (Rabbit: MP-7401 or Mouse: MP-7402, Vector Laboratories) were applied for 30 min, followed by DAB staining (SK-4103, Vector Laboratories). After PBS washes, nuclear staining with hematoxylin was performed, and slides were dehydrated and mounted with DPX for microscopic analysis according to the immunoreactivity scoring system (IRS).

### Cell culture

Human NSCLC cell lines (NCI-H460, NCI-H23, NCI-H1299, A549), and human embryonic renal epithelial cell line (HEK-293T) were derived from the American Type Culture Collection (ATCC). According to the instructions of ATCC, NCI-H460, NCI-H23, NCI-H1299, and A549 cell lines were cultured with RPMI-1640 Medium (31800022, Gibco, Waltham, MA, USA) and HEK-293T cell lines were cultured with DMEM medium (12800017, Gibco, Waltham, MA, USA). The complete growth medium used for culture contains 10% fetal bovine serum (10270106, Gibco, Waltham, MA, USA) and 1% Antibiotic-Antimycotic (100X) (15240062, Gibco, Waltham, MA, USA).

### Quantitative real-time PCR

To determine the relative mRNA expression level of each gene, real-time PCR (qPCR) was performed using TB Green Premix Ex Taq II (RR820, Takara, Japan). To distinguish different isoforms of FOXP3, the primers confined to exon 3 were used to detect FOXP3FL, and the ones that span the junction of exon 2 and exon 4 were chosen to target FOXP3Δ3. β-actin was used as an endogenous reference gene for each experiment. Relative mRNA expression levels were calculated by the 2^−ΔΔCt^ method: ΔΔCT = ΔCT (target sample) −ΔCT (reference sample) [37]. Primers used in the quantitative real-time PCR (Q-PCR) assay are listed in Table S1.

### Immunoblot

Protein extraction was performed to extract proteins from tissues and cell lines using RIPA lysis buffer. Protein quantification was performed using DC Protein Assay (5000112, Bio-Rad, Hercules, CA, USA), then denatured with loading dye at 95°C for 10 min. Twenty micrograms of protein were loaded in SDS–PAGE for electrophoresis and then transferred to PVDF membranes (1620177, Bio-Rad, Hercules, CA, USA). In the next step, membranes were incubated with primary and secondary antibodies. At last, the membrane was applied with surfaceClarity™ Western ECL Substrate (1705060, Bio-Rad, USA) and exposed to autoradiography film Kodak (Rochester, NY, USA) in dark room or imaged with a ChemiDoc™ Imaging Systems (Bio-Rad, Hercules, CA, USA).

Primary antibodies: anti-FOXP3Δ3 (1:3000, Novus Biological, NBP2-24953), anti-FOXP3 (1:1000, Cell signaling), anti-EGFR (1:1000, Cell signaling), anti-pEGFR (1:1000, Cell signaling), anti-KRAS (1:1000, Cell signaling), anti-STAT3 (1:1000, Cell signaling), anti-pSTAT3 (1:1000, Cell signaling), anti-Snail (1:1000, Santa Cruz), anti-Slug (1:1000, Cell signaling), anti-Vimentin (1:10,000, Santa Cruz), anti-GAPDH (1:10,000, Santa Cruz), anti-Lamin B1 (1:1000, Cell signaling). Secondary antibodies in this study included anti-mouse IgG-HRP (1:4000, Cell Signaling) and anti-rabbit IgG-HRP (1:4000, Cell Signaling).

### Plasmids

pBABE-puro was a gift from Hartmut Land & Jay Morgenstern & Bob Weinberg (Addgene Plasmid No. 1764); EGFR WT (Addgene Plasmid No. 11011), pBabe EGFR (T790M) (Addgene Plasmid No. 32070), pBabe EGFR (Addgene Plasmid No. 32062), EGFR L858R (Addgene Plasmid No. 11012), and EGFR G719S (Addgene Plasmid No. 11013) were gifts from Matthew Meyerson. pBabe-Kras Wt (Addgene Plasmid No. 75282), pBabe-Kras G12D (Addgene Plasmid No. 58902), and pBabe-Kras G12C (Addgene Plasmid No. 58901) were gifts from Channing Der. pBABE-Puro-KRas*G12V was a gift from Christopher Counter (Addgene Plasmid No. 46746).

Short hairpin RNAs (shRNAs) targeting FOXP3Δ3 were designed using The Genetic Perturbation Platform and inserted into the pLKO.1-puro vector backbone (a gift from Bob Weinberg, Addgene Plasmid No. 8453). The targeting sequence, CATCGCAGCTGCAGCTCTCAA, was positioned at the junction of FOXP3 exons 2 and 4. Four shRNAs were initially designed to target FOXP3Δ3, but only one proved effective. FOXP3Δ3sh1 and sh2 mentioned in this study are 2 clones of this functional shRNA.

Overexpression plasmids pcDNA3.1-FOXP3FL, pcDNA3.1-FOXP3Δ3, and pcDNA3.1-HA-STAT3 were generated in our lab; pLV-3xFlag-Puro-C, pLV-FOXP3-full-length-Puro-C, and pLV-FOXP3-delta3-Puro-C were purchased from Inovogen Tech. Co. (Chongqing, China); psPAX2 and pMD2.G (gift from Didier Trono; Addgene Plasmid No. 12260; No. 12259) were used as virus packaging plasmids.

### The generation of stably transfected cells

HEK-293T cells were seeded in 6-well plates, and transfection was conducted using Lipofectamine™ 3000 Reagent. Viral supernatant was collected at 24th hour and 48th hour, respectively.

Cells to be infected were seeded in 6-well plates, and the viral supernatant was added with polybrene (2 μg/mL). Culture medium containing the virus was removed after 24 h, and infection was completed at the 48th hour after virus addition. Afterward, cells were selected by puromycin (A1113803, Gibco, MA, USA) at the appropriate concentration for 5 days to generate stable cell lines.

### Cell proliferation assay

Cell proliferation was determined by MTT assay. Stable cells were seeded into 96-well plates at a density of 1000 cells per well in 200 µL culture medium. After 4 h, cells adhered, and 20 µL MTT (3-(4,5-dimethylthiazol-2-yl)-2,5-diphenyltetrazolium bromide, M5655, Sigma-Aldrich, St. Louis, MO, USA) solution (5 mg/mL in PBS) was added and incubated for 3 h at 37°C. After incubation, supernatants were discarded, and 200 µL DMSO (Dimethyl Sulfoxide) was added to dissolve the crystal by pipetting up and down. The absorbance was detected at 570/630 nm in a plate reader.

### Colony formation assay

NSCLC cells were trypsinized and resuspended in culture medium. Cells were then counted by a hemacytometer and seeded in 6-well plates at a density of 1000 cells per well. After 7–10 days of incubation, the supernatant was removed, and 1 mL of 1% Crystal Violet solution (dissolved in 25% methanol) was added for staining. The plate was incubated at 37°C for 30 min, followed by washing with PBS. After air drying, images were acquired by a scanner.

### TUNEL assay

TUNEL assay was performed to detect the apoptosis status of cells under different treatments using *In situ* Cell Death Detection Kit (11684817910, Sigma-Aldrich, St. Louis, MO, USA). Cells were treated with 50 μg/mL DDP (cisplatin) for 24 h and then seeded onto coverslips. After treatment, cells were fixed with 4% Paraformaldehyde and then blocked with 3% H_2_O_2_. In the next step, cells were treated with freshly prepared Permeabilization. Thereafter, cells were incubated with 50 µL TUNEL reaction mixture (5 µL Enzyme solution, 45 µL Label Solution) for 1 h in dark. A negative control that was incubated with 50 µL/well Label Solution was set up. After incubation, slides were mounted by ProLong™ Gold Antifade Mountant with DAPI (P36935, Invitrogen, MA, USA) and then observed and photographed under a fluorescence microscope.

### Wound healing assay

Stable cell lines were seeded in 6-well plates, and when reaching 80% confluence, a straight scratch was created using 200 µL yellow tips. Then, the plates were washed by PBS to remove exfoliated cells and incubated in medium containing 1% FBS in order to inhibit proliferation. Images were photographed immediately after scratching as 0 and 24 h later. Changing the gap size was used to evaluate the migration ability of cells.

### Cell invasion assay

Before the experiment, Falcon^®^ Cell Culture Inserts 8.0 µm (353097, Corning, NY, USA) were coated with 100 µL 20% Matrigel Matrix (Corning, NY, USA) and incubated at 37°C for 4 h. Cells were trypsinized and resuspended in serum-free culture medium. When reaching 80% confluence, cells were counted by a hemocytometer. 5 × 10^5^/200 µL cells were seeded into the upper chamber of the inserts, and 600 µL complete culture medium was filled into the lower chamber. After 24-h incubation, inserts were stained with 1% crystal violet solution for 30 minutes. Cells that passed through the membrane were photographed and counted under a microscope.

### Sphere formation assay

Stable cells were trypsinized and resuspended in Cancer Stem Premium (20141-500, ProMab, CA, USA) culture medium. When reaching 80% confluence, cells were counted by a hemocytometer. Cells were seeded into 6-well ultra-low attachment plates (3471, Corning, Somerville, MA, USA) at a density of 5000 per well in a 2 mL stem cell culture medium. After 10 days, cells were photographed under a microscope, and cell clusters that were ≥ 50 µm were counted as spheres.

### Coimmunoprecipitation assay

To detect the binding ability of STAT3 and different FOXP3 isoforms, HEK-293T cells were transfected with pcDNA3.1-HA-STAT3 (constructed in our lab) and pLV-FOXP3-full-length-Puro-C or pLV-FOXP3-delta3-Puro-C. Forty-eight hours after transfection, cells were lysed and sonicated. The lysate was incubated with anti-FLAG^®^ M2 Magnetic Beads (Sigma-Aldrich, St. Louis, MO, USA) or Pierce™ Anti-c-Myc Magnetic Beads (Thermo Fisher Scientific, MA, USA) and washed with PBS. Then beads were denatured, proteins eluted in the supernatant were used for the Western-blot assay.

### Xenograft animal assay

Four-week-old female nude mice were obtained from the Laboratory Animal Service Center (LASC) of CUHK and kept in the animal room according to the guidelines of the Department of Health HKSAR Government. 2.5 × 10^6^ of H460-PLKO.1, H460-FOXP3Δ3-sh1, and H460-FOXP3Δ3-sh2 stable cells were subcutaneously injected into the back of nude mice, respectively, with a 1 mL sterile syringe. The tumor size was measured every 3 days by a vernier caliper, and tumor volume was calculated by *V *= 1/2 (length × width^2^). Mice were sacrificed if the diameter of the tumor reached 12 mm or at the end of the experiment. Tumors were isolated, rinsed in PBS, and fixed in formalin for IHC analysis. Animal experiments were approved by the Animal Ethics Committee of the Chinese University of Hong Kong.

### Statistical analysis

Statistical analysis was performed with the help of SPSS version 16.0 (SPSS, Chicago, IL, USA) and Graph Pad Prism version 7.0. One-way ANOVA with repeated measures was used to compare quantitative variables such as the number of colonies formed, apoptotic cells, and cells migrated or invaded between three groups. The Student’s *t*-test was used to compare two groups. When analyzing the clinicopathologic features in patients with high and low expressing FOXP3Δ3, the *t*-test was used to analyze quantitative variables such as age and tumor size. Pearson’s chi-squared test was applied to measure qualitative variables like gender, smoking status, and M/N Status. The Mann–Whitney *U* test was employed to analyze ordinal variables such as stage and T status. Kaplan–Meier plots were used for overall survival (OS) rates. *P-*value < 0.05 was considered statistically significant.

## Supplementary Material

lnag024_Supplementary_Data

## Data Availability

All data needed to evaluate the conclusions in the paper are present in the paper and/or the Supplementary Materials. Artificial intelligence (AI) use statement During manuscript preparation, we used an AI-based language assistant (Perplexity, powered by GPT-5.1) to help refine wording, improve clarity, and check grammar. All scientific content, study design, data analysis, and interpretation were conceived and verified by the authors, who take full responsibility for the accuracy and integrity of the work. Consent for publication statement The authors declare their agreement to publish.
