# Supplementary material for: Tumor cell-specific exon 3-deleted FOXP3 isoform promotes growth and metastasis via STAT3 in non-small cell lung cancer
Source: Life Med. 2026 Jul 7;5(4):lnag024. doi: 10.1093/lifemedi/lnag024 (PMC13493058; doi:10.1093/lifemedi/lnag024)
Supplement: Supplementary file 1 [file lnag024_supplementary_data.zip › Supplementary_Figures _PE .docx]

**Tumor cell–specific exon 3‑deleted FOXP3 isoform promotes growth and metastasis via STAT3 in non‑small cell lung cancer**

Jia Peng^2,^^#^, Yi Liu^4,#^, Zhongqin Gong^2^, Ming-yue Li^5^, Hao Jia^6^, Xueqing Zhou^1^, Calvin S.H. Ng^2,*^, George G. Chen^2,3,*^, Shucai Yang^1,*^

^1^Department of Laboratory Medicine, Pingshan Hospital, Southern Medical University (People’s Hospital of Shenzhen Pingshan), Shenzhen 518118, China

^2^Department of Surgery, The Chinese University of Hong Kong, Prince of Wales Hospital, Hong Kong 00000, China

^3^Department of Otorhinolaryngology, Head and Neck Surgery, The Chinese University of Hong Kong, Prince of Wales Hospital, Hong Kong 00000, China

^4^School of Ocean and Tropical Medicine, Guangdong Medical University, Zhanjiang 524023, China

^5^GuangZhou Laboratory, No.9 XingDaoHuanBei Road, Guangzhou International Bio Island, Guangzhou 510005, China

^6^Department of Thyroid and Breast Surgery, Peking University Shenzhen Hospital, Shenzhen 518036, China

^#^These authors contributed equally to this work.

^*^Correspondence: shucaiyang@link.cuhk.edu.hk (S.Y.); gchen@cuhk.edu.hk (G.G. C.); calvinng@surgery.cuhk.edu.hk (C.S.H. Ng)

**Figure. S1** (A) Violin plot showing the expression distribution of annotated FOXP3 transcript variants in LUAD based on TCGA data analyzed using GEPIA2. FOXP3-201 (ENST00000376199; corresponding to FOXP3Δ3) and FOXP3-001 (corresponding to FOXP3FL). (B) Independent validation of FOXP3 transcript variant expression in LUAD using UCSC Xena (C) Kaplan–Meier analysis of overall survival based on the FOXP3 exon-skipping event ES89118 (corresponding to FOXP3Δ3) in rectal adenocarcinoma (READ) from the UCSC Xena database. Log-rank *P* = 2.8 × 10^−2^. (D) Kaplan–Meier analysis of disease-free interval (DFI) based on FOXP3 ES89118 in sarcoma (SARC) from UCSC Xena. Log-rank *P* = 1.5 × 10^−2^. (E) Kaplan–Meier analysis of disease-free interval (DFI) based on FOXP3 ES89118 in thyroid carcinoma (THCA) from UCSC Xena. Log-rank P = 4.36 × 10^−2^.
